# Anti-neuroinflammatory effects of alkaloid-enriched extract from *Huperzia serrata* on lipopolysaccharide-stimulated BV-2 microglial cells

**DOI:** 10.1080/13880209.2022.2159450

**Published:** 2023-01-08

**Authors:** Thu Kim Dang, Seong-Min Hong, Vui Thi Dao, Phuong Thi Thu Tran, Hiep Tuan Tran, Giang Hoang Do, Thanh Nguyen Hai, Hang Thi Nguyet Pham, Sun Yeou Kim

**Affiliations:** a Department of Clinical Pharmacy, University of Medicine and Pharmacy, Vietnam National University, Hanoi, Vietnam; b College of Pharmacy, Gachon University, Incheon, Republic of Korea; c HaNoi University of Pharmacy, Hanoi, Vietnam; d Department of Life Sciences, Vietnam Academy of Science and Technology, University of Science and Technology of Hanoi, Hanoi, Vietnam; e Faculty of Pharmacy, PHENIKAA University, Hanoi, Vietnam; f Centre for Research and Technology Transfer, Hanoi, Vietnam; g National Institute of Medicinal Materials, Hanoi, Vietnam; h Gachon Institute of Pharmaceutical Science, Gachon University, Incheon, Republic of Korea

**Keywords:** Huperzine A, neurodegenerative diseases

## Abstract

**Context:**

Alkaloid-enriched extract of *Huperzia serrata* (Thunb.) Trevis (Lycopodiaceae) (HsAE) can potentially be used to manage neuronal disorders.

**Objective:**

This study determines the anti-neuroinflammatory effects of HsAE on lipopolysaccharide (LPS)-stimulated BV-2 microglial cells and the underlying mechanisms.

**Materials and methods:**

BV-2 cells were pre- or post-treated with different concentrations of HsAE (25-150 µg/mL) for 30 min before or after LPS induction. Cell viability was assessed using a 3-(4,5-dimethylthiazol-2-yl)-2,5-diphenyl tetrazolium bromide assay and no cytotoxicity was found. Nitric oxide (NO) concentration was determined using Griess reagent. The levels of prostaglandin E2 (PGE2), tumor necrosis factor (TNF)-α, interleukin (IL)-1β, and IL-6 were determined using enzyme-linked immunosorbent assay. The levels of inducible nitric oxide synthase (iNOS) and cyclooxygenase (COX)-2 and the phosphorylation of mitogen-activated protein kinase (MAPK) were analyzed using western blotting.

**Results:**

HsAE reduced LPS-induced NO production with half-maximal inhibitory concentration values of 99.79 and 92.40 µg/mL at pre- and post-treatment, respectively. Pre-treatment with HsAE at concentrations of 50, 100, and 150 µg/mL completely inhibited the secretion of PGE2, TNF-α, IL-6, and IL-1β compared to post-treatment with HsAE. This suggests that prophylactic treatment is better than post-inflammation treatment. HsAE decreased the expression levels of iNOS and COX-2 and attenuated the secretion of pro-inflammatory factors by downregulating the phosphorylation of p38 and extracellular signal-regulated protein kinase in the MAPK signaling pathway.

**Discussion and Conclusions:**

HsAE exerts anti-neuroinflammatory effects on LPS-stimulated BV-2 cells, suggesting that it may be a potential candidate for the treatment of neuroinflammation in neurodegenerative diseases.

## Introduction

Alzheimer’s disease (AD) is an age-related, progressive, neurodegenerative disease that impairs memory and cognitive functions (Essa et al. 2012). Neuroinflammation may initiate neurodegenerative diseases, such as AD (Liu et al. 2022) and Parkinson’s disease (PD) (Grotemeyer et al. 2022). Inflammation is regulated by several factors, such as cytokines, chemokines, and products released by glial cells (such as microglia and astrocytes), endothelial cells, and immune cells (Kwon and Koh 2020). Among these factors, microglia, which are innate immune cells of the central nervous system (CNS), mainly participate in neuroinflammation and perform various neuroimmunological functions under normal or pathological conditions (Prinz et al. 2021). Inflammation associated with the progression of AD and PD may be induced by different cytokines, such as the tumor necrosis factor-α (TNF-α), interleukin (IL)-6, IL-12, and IL-18 (Calsolaro and Edison 2016). Microglial cells play an important role in the maintenance of neuronal homeostasis, but external stimuli, such as lipopolysaccharides (LPS), can cause the cells to change microglia, a type of resident immune cells within CNS, from normal to toxic microglial M1 phenotype and induce the secretion of pro-inflammatory mediators, which initiate several major cellular responses (Qiu et al. 2022). Activation of microglia triggers the secretion of pro-inflammatory mediators and pro-inflammatory cytokines, such as prostaglandin E2 (PGE2), IL-6, IL-1β, and TNF-α, and contributes to the pathogenesis of neuroinflammation, which can worsen brain damage (Surace and Block 2012; Kelley et al. 2019). Therefore, identifying novel anti-neuroinflammatory agents that can inhibit neurodegenerative diseases, such as AD, is very important. *Huperzia serrata* (Thunb.) Trevis (Lycopodiaceae) is a plant that is frequently found in Oceania, Asia, and Central America (Huang and He 2010; Ferreira et al. 2016). *H. serrata* is used to manage neuromuscular or cardiovascular diseases or disorders, including contusions, fever, and schizophrenia, *via* the inhibition of cholinesterase activity (Ferreira et al. 2016). Lycopodium alkaloids, such as huperzine A, are the main bioactive constituents of this plant (Luo et al. 2010). Huperzine A is an active acetylcholinesterase inhibitor isolated from *H. serrata* (Wang et al. 2006). It is used to prevent the breakdown of acetylcholine, which is essential for learning and memory. Moreover, previous studies reported that huperzine A attenuates neuroinflammation and oxidative stress markers in traumatic brain injury models. However, the anti-neuroinflammatory effects of *H. serrata* extract (HsE), particularly the alkaloid-enriched extract of *H. serrata* (HsAE), have not yet been investigated. In this study, we investigated the potential anti-neuroinflammatory effects of HsAE on LPS-stimulated BV-2 microglial cells and its neuroprotective effects on inflamed BV-2 microglial cells and N2a neurons, and determined the underlying mechanisms.

## Materials and methods

### Reagents and chemicals

Dulbecco’s modified Eagle’s medium (DMEM), fetal bovine serum (FBS), and penicillin/streptomycin (supplier) were obtained from Gibco (Grand Island, NY, USA). LPS of *Escherichia coli* and 3-(4,5-dimethylthiazol-2-yl)-2,5-diphenyl tetrazolium bromide (MTT) were obtained from Sigma (St. Louis, MO, USA). N-monomethyl-l-arginine (L-NMMA; Abcam), iNOS (Bioscience), COX-2 (Santa Cruz), and α-tubulin (Sigma-Aldrich) were also purchased. p38 (9212S), phospho (p)-p38 (9211S), extracellular signal-regulated protein kinase (ERK; 9102S), p-ERK (9101S), c-jun N-terminal kinase (JNK; 4671S), and p-JNK (9251S) were purchased from Cell Signaling (Beverly, MA, USA).

### Plant material extraction

As described in a previous article (Dang et al. 2022), HsAE, the alkaloid fraction of the total ethanol extract (HsE), was extracted and one of the main bioactive compounds in it was huperzine A. Aerial parts of *H. serrata* were collected from Da Lat, Lam Dong province, Vietnam, in September, 2019. The plant was identified by Master Nguyen Quynh Nga and Master Phan Van Truong at the Department of Medicinal Resources, National Institute of Medicinal Materials (NIMM), Hanoi, Vietnam. A voucher specimen (No. DL-100919) was deposited in the NIMM. The alkaloid-enriched extract was prepared as described in our previous article. In brief, dried *H. serrata* samples were extracted in 90% EtOH at room temperature, and then the extract was filtered and evaporated under vacuum. The residue was re-dissolved in the solution of 5% HCl and partitioned three times with ethyl acetate. Next, the acidic layer was adjusted to pH 10-11 by 20% NaOH and partitioned three times by dichloromethane. The organic layers were collected and evaporated to yield the HsAE.

### Ultra-performance liquid chromatography-tandem mass spectrometry (UPLC-MS/MS) analysis for the identification of HsAE components

Extracts were analyzed on a SCIEX X500R QTOF mass spectrometer (SCIEX, US), and separation was performed using Phenomenex Kinetex C^18^, (2.1 × 100 mm, 2.6 µm) at 40 °C. The mobile phases consisted of phase A (0.05% formic acid in water) and phase B (0.05% formic acid in acetonitrile) at a flow rate of 300 µL/min. A gradient of 10–70% B for 15 min followed by 100% B for 5 min was used. MS spectra were detected in both positive and negative ion modes. MS parameters were set as follows: air curtain gas CUR: 35 psi; IS voltage: +4500 V (positive mode) or −4500 V (negative mode); source temperature: 550 °C; cone voltage: +80 V (positive mode) or −80 V (negative mode); atomizing gas: 1,55 psi; auxiliary gas: 2,55 psi. The spectra were scanned over the range of 100–2000 Da.

### Cell culture

The BV-2 mouse microglial cell line was obtained from Dr. E. Choi at Korea University (Seoul, South Korea), and the murine neuroblastoma cell line (N2a) was originally obtained from the American Type Culture Collection (Manassas, VA, USA). Each cell was cultured in DMEM supplemented with 10% FBS and 1% penicillin/streptomycin at 5% CO_2_ and 37 °C.

### Cell viability assay

Cells were cultured in a 96-well plate (4 × 10^4^ cells/well) and treated with different sample concentrations for 30 min before or after LPS (100 ng/mL) induction. The cells of the control group were neither treated with HsAE nor induced with LPS. After incubation for 24 h, the medium was removed, and MTT solution was added to the cells at a final concentration of 0.5 mg/mL. The cells were incubated at 37 °C for 2 h. The medium was carefully removed, and 100 µL of dimethyl sulfoxide was added to each well. The absorbance was measured using a microplate reader (Bio-Rad, USA) at a wavelength of 570 nm (Subedi et al. 2019).

### Nitric oxide (NO) production

BV-2 cells were seeded in a 96-well plate at a density of 4 × 10^4^ cells/well and incubated overnight. Seeded cells were pre- or post with different concentrations of HsAE and huperzine A for 30 min before or after LPS (100 ng/mL) induction. Notably, L-NMMA (25 µM), a well-known NO synthase (NOS), was used as a positive control (Reif and McCreedy 1995). After 24 h of treatment, 50 μL of conditioned medium from each well was transferred to a new 96-well plate and mixed with an equal volume of Griess reagent including 1% sulfanilamide and 0.1% *N*-(1-naphthyl) ethylenediamine dihydrochloride. Sodium nitrite solution was used as a standard to calculate nitrite concentrations, which were measured at a wavelength of 540 nm (Subedi et al. 2019).

### Measurement of PGE2, TNF-α, IL-1β, and IL-6 levels

PGE2, TNF-α, IL-1β, and IL-6 levels were measured using an enzyme immunoassay. BV-2 cells seeded at 5 × 10^4^ cells/well and pre- or post-treated with HsAE at various concentrations for 30 min before or after LPS (100 ng/mL) induction, and incubated for 24 h. The next day, the culture medium was collected and centrifuged at 5,000 × *g* for 10 min. These cytokines were evaluated using kits (R&D Systems, Minneapolis, MN, USA), according to the manufacturer’s protocol.

### Western blotting analysis

BV-2 cells were seeded at a density of 1 × 10^6^ cells/plate and treated with or without LPS (100 ng/mL) in the presence or absence of HsAE at various concentrations. After treatment for 30 min, cells were washed and lysed using PRO-PREP solution (iNtRON, Seoul, Korea) containing protease and phosphatase inhibitors. Protein estimation was performed using the Bradford assay, and proteins (30 µg) were separated using sodium dodecyl sulfate-polyacrylamide gel electrophoresis and transferred to nitrocellulose membranes. The membranes were blocked with 5% skim milk in Tris-buffered saline containing 0.1% Tween 20 (TBST) at room temperature for one hour and incubated with primary antibodies (1:1000) at 4 °C overnight. The next day, membranes were incubated with secondary antibodies (1:2000) in TBST with 5% skim milk. Protein expression was visualized using the ChemiDoc XSR + System and Image lab 6.1 software (Bio-Rad Laboratories, USA) (Subedi et al. 2019).

### Neuronal cell viability measurement

N2a cell line was used to examine the apoptotic neuronal cell viability using conditioned medium (CM) in LPS-stimulated BV-2 cells treated with HsAE (Subedi et al. 2017). To measure neuronal cell viability, BV-2 cells were treated with different concentrations of 100 ng/mL LPS for 30 min in the presence or absence of HsAE. After 24 h, the CM was collected and used to treat N2a cells for 24 h. Cell viability was measured using an MTT assay.

### Statistical analysis

All data are expressed as mean ± standard error of the mean. Statistical comparisons between different treatments were performed using one-way analysis of variance followed by Tukey’s *t*-test as a *post hoc* test using GraphPad Prism 8 (GraphPad Software, Inc., San Diego, CA, USA). Statistical significance was set at *p < 0.05, **p < 0.01, and ***p < 0.001.

## Results

### Chemical profile of compounds in HsAE

**Figure 1.**
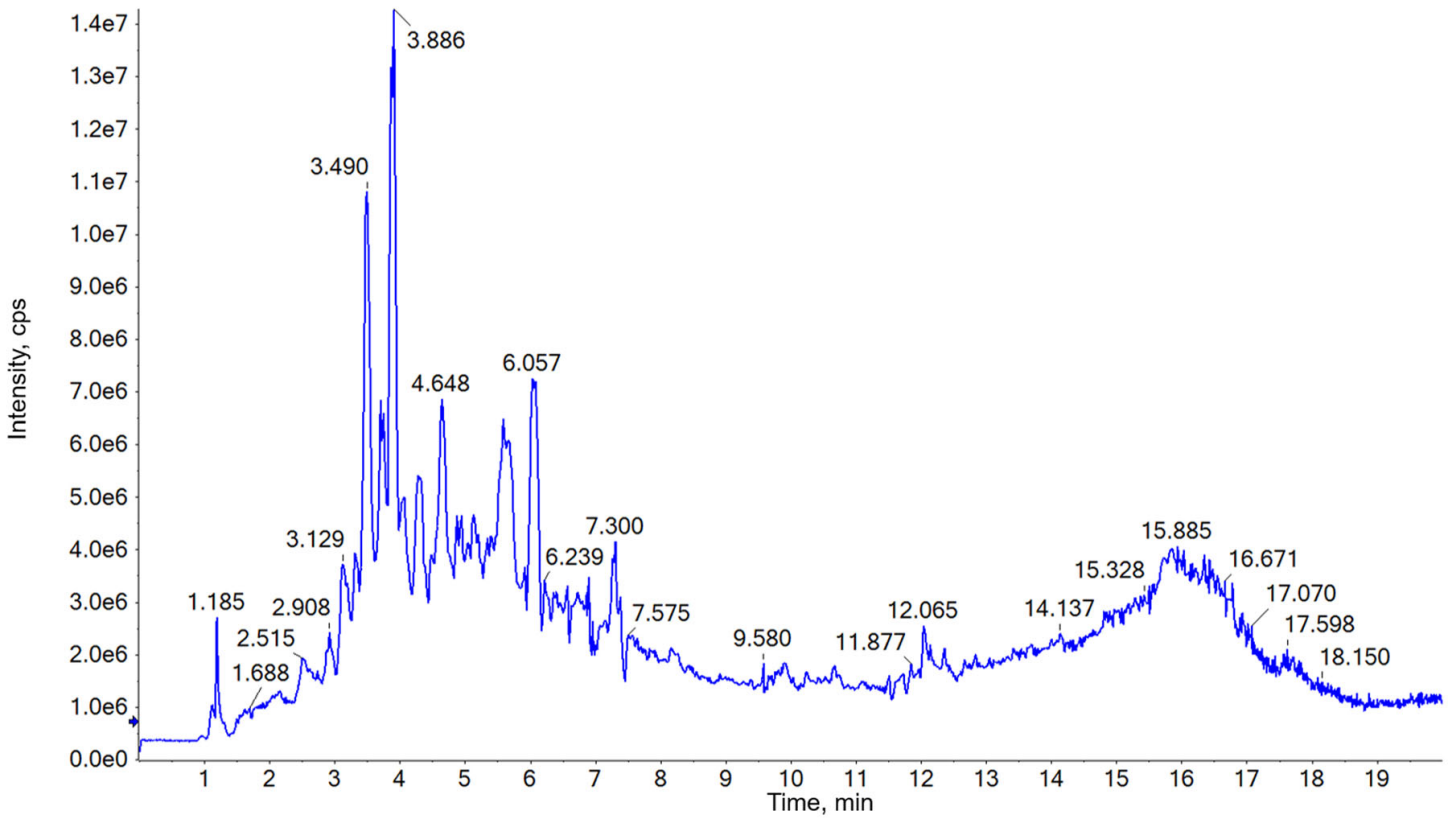
Total ion chromatogram of the alkaloid-enriched extract of *Huperzia serrata* (HsAE) in positive mode.

Chemical profiles of the alkaloids in HsAE were investigated using UPLC-MS/MS analysis. The total ion chromatogram is shown in Figure 1 and the results are given in Table 1.

**Table 1.** Alkaloid identification from the alkaloid-enriched extract of *Huperzia serrata* (HsAE) using ultra-performance liquid chromatography-tandem mass spectrometry (UPLC-MS/MS).

Index	retention time(min)	Adducts	MS^2^ fragments (positive mode)	Identification	Ref.
1	2.91	265 [M + H]^+^	248, 236, 224, 208, 194, 184, 146	Unknown lycodine-type alkaloid	(Shan et al. 2016)
2	3.49	257 [M + H]^+^	240, 212, 198, 184	Huperzine B	(Li et al. 2004; Wu and Gu 2006; Shan et al. 2016)
3	3.89	243 [M + H]^+^	226, 211, 197, 184	Huperzine A	(Li et al. 2004; Wu and Gu 2006; Shan et al. 2016)
4	4.32	278 [M + H]^+^	262, 248, 221	Unknown lycodine-type alkaloid	(Shan et al. 2016)
5	4.65	308 [M + H]^+^	293, 280, 264	Acetylannofoline or its isomer	(Wu and Gu 2006)
6	4.76	279 [M + H]^+^	264 , 162	Huperzine M	(Li et al. 2004)
7	4.84	306 [M + H]^+^	291, 278, 262	Acetylannofoline or its isomer	(Wu and Gu 2006)
8	5.62	309 [M + H]^+^	291, 263, 235	Acetylannofoline or its isomer	(Wu and Gu 2006)
9	6.10	262 [M + H]^+^284 [M + Na]^+^	244	Unknown	
10	7.37	262 [M + H]^+^284 [M + Na]^+^	244	Unknown	

Major compounds were selected based on their retention time (rt) and intensity and identified by comparing their molecular and fragment ions with those of published data. As shown in Table 1, most of the constituents exhibited [M + H]^+^ ions and MS^2^ fragments in the positive mode. Huperzine A (compound **3**), the major phytochemical in *H. serrata*, could be identified at 3.89 min on the chromatogram due to the highest peak intensity and the precursor ion [M + H]^+^ *m/z* 243 as well as the specific fragments, [M + H-NH_3_]^+^ *m/z* 226, [M + H-CH_3_-NH_3_]^+^ *m/z* 211, and [M + H-C_2_H_5_-NH_3_]^+^ *m/z* 197 (Wu and Gu 2006; Shan et al. 2016). Whereas, compound **2**, at 3.49 min, could be determined as huperzine B by comparing its precursor ion [M + H]^+^ *m/z* 257 and MS^2^ fragments at [M + H-NH_3_]^+^ *m/z* 240, [M + H-C_2_H_5_NH_2_]^+^ *m/z* 212, [M + H-C_3_H_7_NH_2_]^+^ *m/z* 198, and [M + H-C_4_H_9_NH_2_]^+^ *m/z* 184 to those in the literature data (Wu and Gu 2006; Shan et al. 2016). MS spectrum of compound **6** (retention time of 4.76 min) exhibited the molecular ion [M + H]^+^ at *m/z* 279, together with MS^2^ fragments at [M + H-CH_3_]^+^ *m/z* 264 and [M + H-CH_3_NH_2_-CH_3_-C_3_H_5_NO]^+^ *m/z* 162. These results are in agreement with the MS data of huperzine M in a previous study (Liu et al. 2017); thus, compound **6** was determined to be huperzine M. Compounds **5**, **7**, and **8** exhibited precursor ions [M + H]^+^ at *m/z* 308, 306, and 309, respectively, and their MS^2^ fragments also showed a highly similar trend. These results revealed the presence of three acetylannofoline isomers, which have been described in previous reports (Wu and Gu 2006).

MS spectrum of compound **1** (retention time of 2.91 min) showed the precursor ion [M + H]^+^ at *m/z* 265, which was the same as that of huperzine K. However, its fragment ions, such as [M + H-NH_3_]^+^ *m/z* 248, [M + H-2CH_3_]^+^ *m/z* 236, [M + H-C_3_H_6_]^+^ *m/z* 224, [M + H-C_3_H_6_NH_2_]^+^ *m/z* 208, and [M + H-C_4_H_8_NH_2_]^+^ *m/z* 194, did not match the reported data for huperzine K. Moreover, fragments at *m/z* 184 and 146 were observed in the MS/MS spectrum of compound **1**. Similarly, compound **4** (retention time of 4.32 min) exhibited [M + H]^+^ at *m/z* 278 and its fragment ions, such as [M + H-NH_3_]^+^ *m/z* 262, [M + H-2CH_3_]^+^ *m/z* 248, and [M + H-C_3_H_6_NH_2_]^+^ *m/z* 221. These were similar to the fragmentation of huperzine B or other lycodine-type alkaloids with a 2-pyridone type (A-ring) and piperidine ring (C-ring) (Shan et al. 2016) but did not match any reported compounds from *Huperzia* species. Thus, compounds **1** and **4** could be novel lycodine-type alkaloids that should be isolated for further structural elucidation. Compounds **9** and **10** could be two isomers as both compounds had a molecular weight of 261 Da due to [M + H]^+^ at *m/z* 262 and [M + Na]^+^ at *m/z* 284 in the MS spectra. [M + H-NH3]^+^ fragment at *m/z* 244 indicates an amino group in the structure of these compounds. This observation did not match the structure of any other compounds with the same molecular weight that were isolated from the genus *Huperzia*. Therefore, these compounds could be interesting targets for further isolation and structure elucidation studies on *H. serrata.*

### HsAE reduces NO production in LPS-induced microglial cells

**Figure 2.**
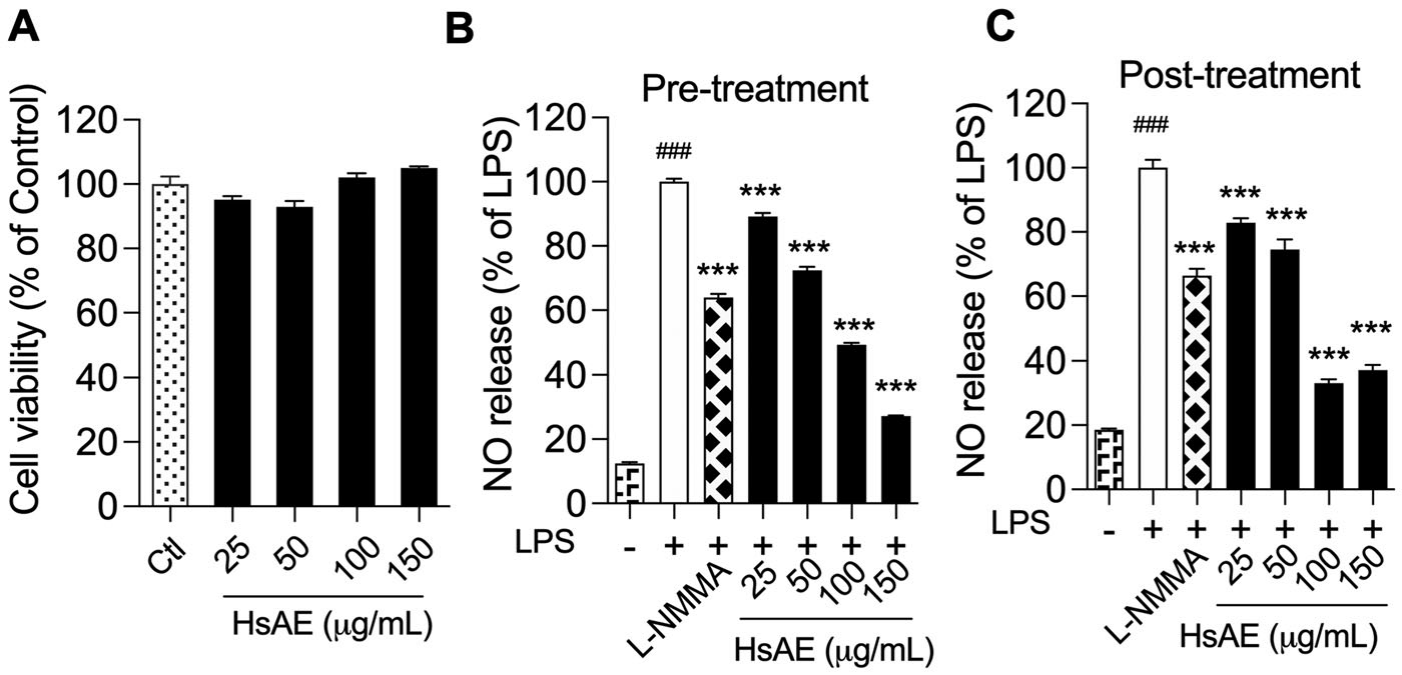
HsAE interacts with microglial cells without cytotoxicity and inhibits inflammation. BV-2 cells were pre- or post-treated with HsAE at concentrations of 25, 50, 100, and 150 µg/mL for 30 min and induced with lipopolysaccharide (LPS; 100 ng/mL) for 24 h. (A) Cytotoxic effect of HsAE on BV-2 cells. (B) Nitrite release upon HsAE pre-treatment. (C) Nitrite release upon HsAE post-treatment. All data are presented as the mean ± standard error of the mean (SEM) (*n* = 3). Statistical differences were analyzed using one-way analysis of variance (ANOVA) followed by Tukey statistical *post hoc* test. *p < 0.05, **p < 0.01, ***p < 0.001 vs. LPS-treated group, ^###^*p* < 0.001 vs. untreated control group.

To verify that HsAE was not toxic to BV-2 cells, an MTT assay was conducted to determine the viability of cells treated with HsAE at concentrations ranging from 25 to 150 µg/mL. HsAE was not cytotoxic at any of the tested concentrations (Figure 2(A)). Therefore, these concentrations were used to investigate the anti-neuroinflammatory effects of HsAE on LPS-stimulated BV-2 cells.

LPS activates BV-2 cells to release large quantities of NO. The effect of HsAE on LPS-induced NO production in BV-2 microglial cells was compared before and after treatment. LPS strongly stimulated NO secretion in BV-2 cells, compared to the unstimulated control, by 8.05- and 5.41-fold, respectively, before and after treatment (Figure 2(B,C)). In the pre-treatment, HsAE at concentrations of 25, 50, 100, and 150 µg/mL significantly reduced NO secretion by 10.87, 27.59, 50.80, and 72.82%, respectively, compared to the LPS group. In the post-treatment, HsAE also significantly impaired LPS-induced NO production at concentrations of 25, 50, 100, and 150 µg/mL by 17.33, 24.63, 66.59, and 62.39%, respectively, compared to the LPS group. HsAE at a concentration of 150 µg/mL decreased NO production by 2.4-fold in the pre-treatment and 1.79-fold in the post-treatment more potently than L-NMMA. Furthermore, the half-maximal inhibitory concentration (IC_50_) values of HsAE and huperzine A showed that the pattern of activity was similar to its effects on NO secretion in both pre- (IC_50_ value of HsAE, 99.79 µg/mL; IC_50_ value of huperzine A, 38.04 µg/mL) and post-treatment (IC_50_ value of HsAE, 92.40 µg/mL; IC_50_ value of huperzine A, 40.23 µg/mL) (Table 2).

**Table 2.** Effects of HsAE and huperzine A on nitric oxide (NO) production in lipopolysaccharide (LPS)-stimulated BV-2 cells.

	IC_50_^a^	Cell *via*bility (%)
Pre-treatment		
HsAE^b^	99.79 µg/mL	105.05 ± 1.18
Huperzine A^c^	38.04 µg/mL	116.19 ± 1.66
L-NMMA^d^	3.28 µg/mL	113.95 ± 6.95
Post-treatment		
HsAE^b^	92.40 µg/mL	105.05 ± 1.18
Huperzine A^c^	40.23 µg/mL	90.59 ± 5.19
L-NMMA^d^	4.52 µg/mL	97.29 ± 2.15

^a^IC_50_ value was defined as the concentration (µg/mL or µM) that caused 50% inhibition of NO production in LPS-stimulated BV-2 cells.

^b,c,d^Cell viability after treatment with 150 µg/mL HsAE, 250 µM huperizne A, and 25 µM L-NMMA was assessed using the 3-(4,5-dimethylthiazol-2-yl)-2,5-diphenyl tetrazolium bromide (MTT) assay and expressed as a percentage. All data are presented as the mean ± standard error of the mean (SEM) (*n* = 3).

### HsAE suppresses the production of pro-inflammatory cytokines in LPS-stimulated microglial cells

**Figure 3.**
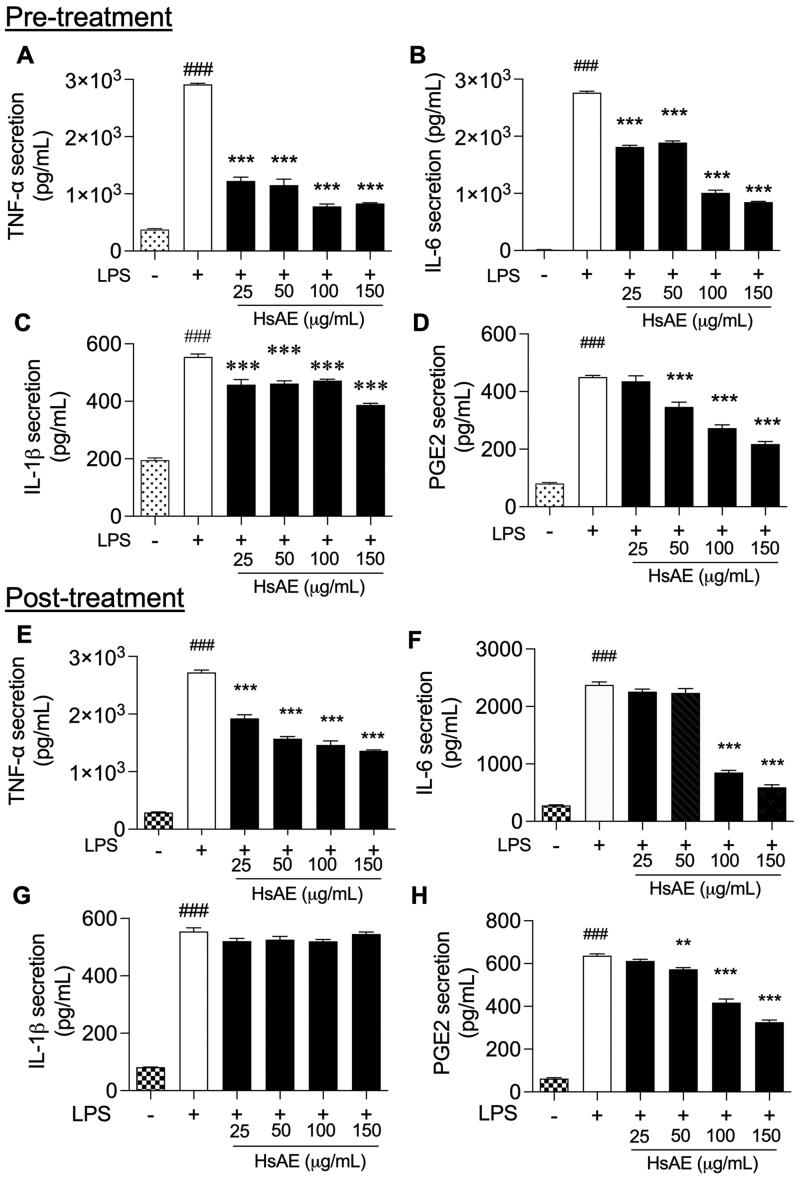
HsAE inhibits pro-inflammatory cytokine secretion in LPS-stimulated BV-2 cells. BV-2 cells were pre- or post-treated with HsAE at concentrations of 25, 50, 100, and 150 µg/mL for 30 min and induced with LPS (100 ng/mL) for 24 h. (A–D) Secretion of tumor necrosis factor (TNF)-α, interleukin (IL)-6, IL-1β, and prostaglandin E2 (PGE2) after HsAE pre-treatment. (E–H) Secretion of TNF-α, IL6, IL-1β, and PGE2 after HsAE post-treatment. All data are presented as the mean ± SEM (*n* = 3). Statistical differences were analyzed using one-way ANOVA followed by Tukey statistical *post hoc* test. *p < 0.05, **p < 0.01, ***p < 0.001 vs. LPS-treated group, ^###^*p* < 0.001 vs. untreated control group.

After LPS treatment for 24 h, TNF-α, IL-6, IL-1β, and PGE2 release was significantly increased compared to that in the unstimulated control. HsAE significantly reduced the production of each cytokine compared to the LPS-stimulated pre-treatment (Figure 3(A–D)). In the post-treatment, HsAE at all concentrations significantly decreased the production of TNF-α compared to the LPS-treated group (Figure 3(E)). A significant reduction in IL-6 secretion occurred only at HsAE concentrations of 100 and 150 µg/mL (Figure 3(F)), whereas PGE2 secretion occurred at HsAE concentrations of 50, 100, and 150 µg/mL (Figure 3(H)). HsAE did not significantly affect IL-1β secretion under post-treatment conditions (Figure 3(G)).

### HsAE inhibits the expression of COX-2 and iNOS in LPS-stimulated microglial cells

**Figure 4.**
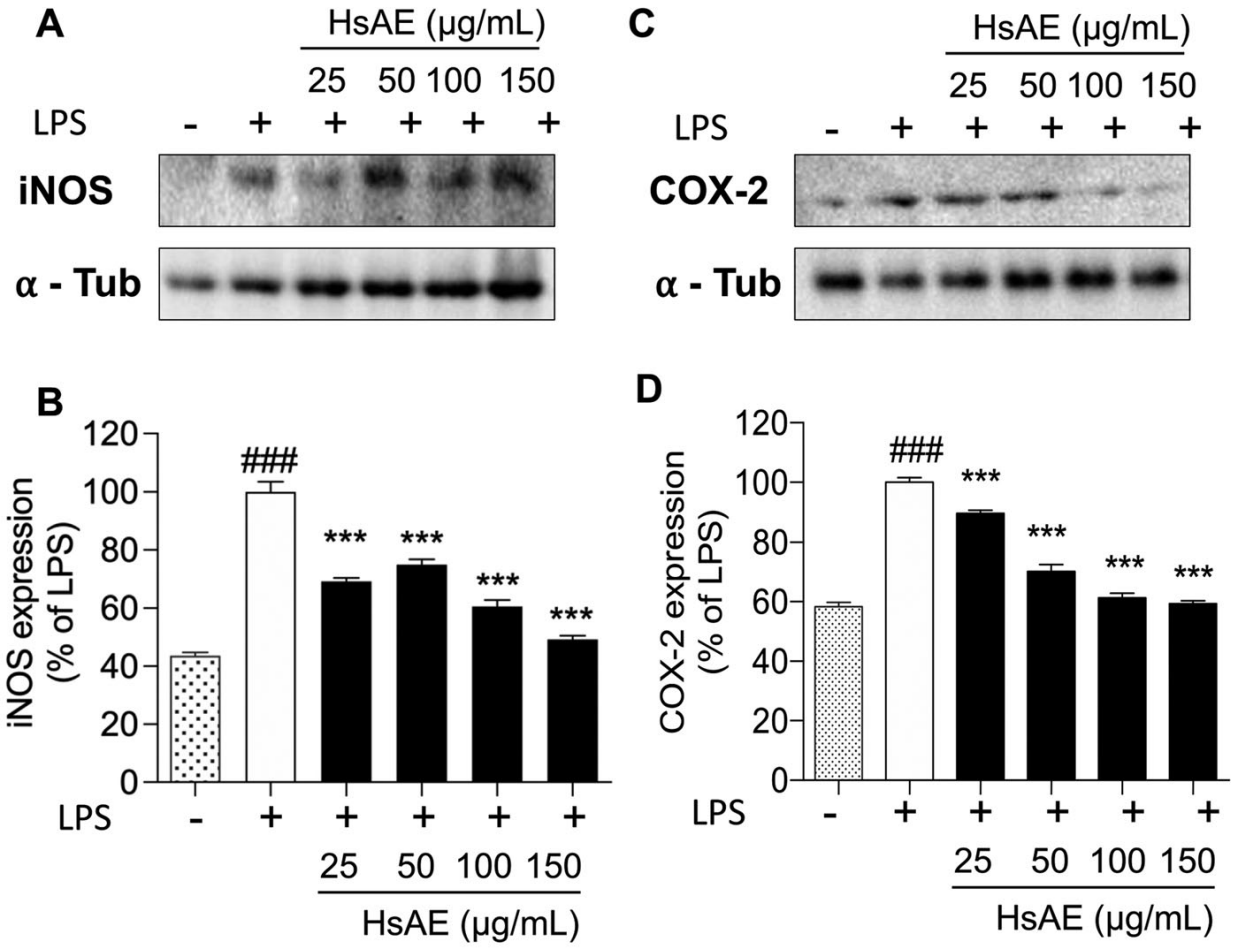
Effects of HsAE on the expression levels of inducible nitric oxide synthase (iNOS) and cyclooxygenase 2 (COX-2) in LPS-stimulated BV-2 cells. (A,C) Densitometric analysis of iNOS and COX-2 proteins as a percentage of LPS-treated group (set as 100%). α-Tubulin was used as the loading control. (B,D) Quantification of (A,C). All data are presented as the mean ± SEM (*n* = 3). Statistical differences were analyzed using one-way ANOVA followed by Tukey statistical *post hoc* test. *p < 0.05, **p < 0.01, ***p < 0.001 vs. LPS-treated group, ^###^*p* < 0.001 vs. untreated control group.

To investigate whether the inhibition of NO and PGE2 production by HsAE in LPS-stimulated BV-2 cells was associated with the levels of iNOS and COX-2, we determined the expression levels of these proteins using western blotting analysis. We found that treatment with HsAE reduced the expression levels of iNOS by 69.10 ± 1.22, 74.87 ± 1.79, 60.63 ± 2.20, and 49.22 ± 1.30%, and COX-2 by 89.67 ± 1.13, 70.17 ± 2.30, 61.33 ± 1.49, and 59.40 ± 0.95% at concentrations of 25, 50, 100, and 150 µg/mL, respectively, compared with the LPS-stimulated group (Figure 4(B,D)).

### HsAE inhibits MAPK signaling in LPS-stimulated microglial cells

**Figure 5.**
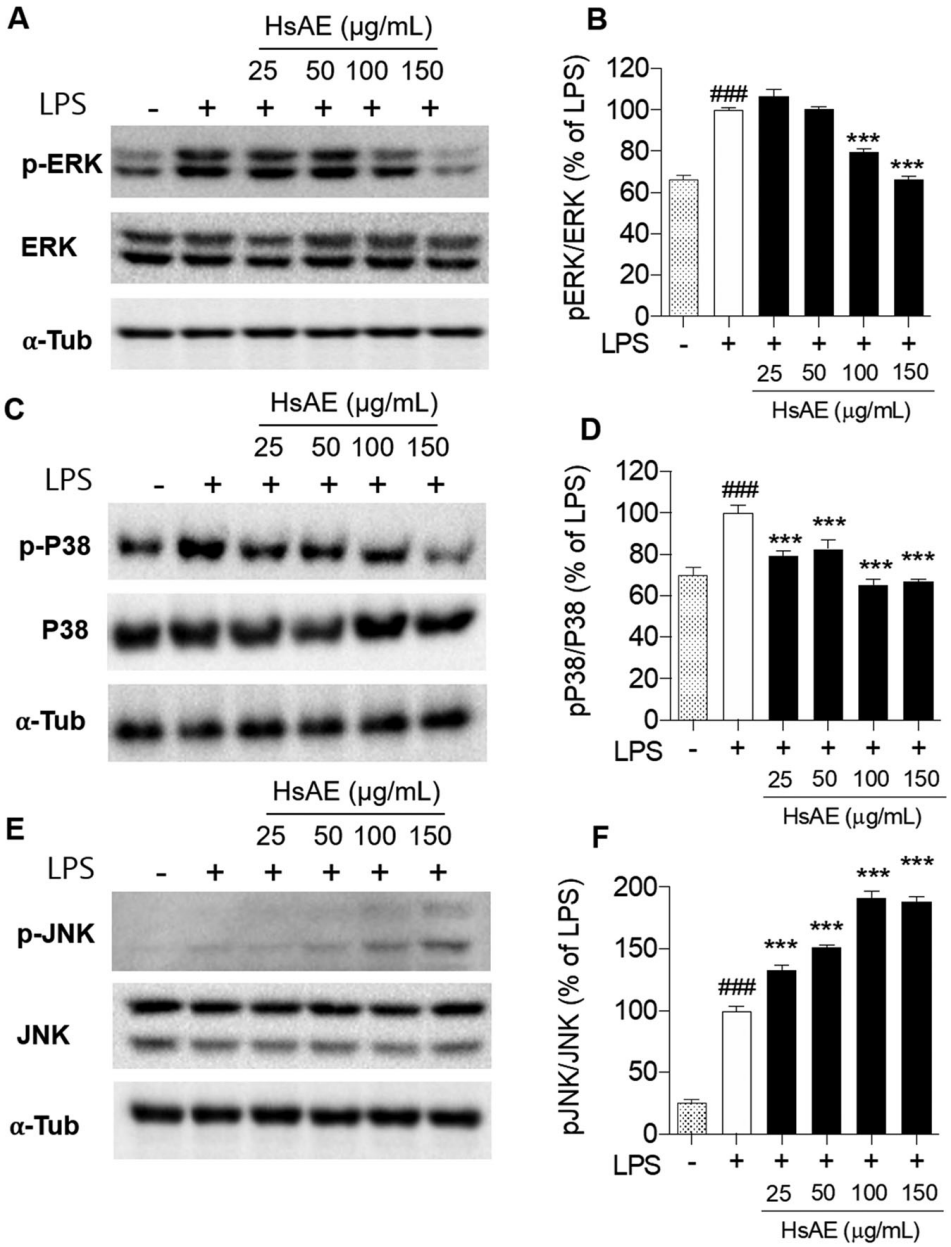
Effect of HsAE on LPS-induced mitogen-activated protein kinase (MAPK) signaling in BV-2 cells. (A, C, E) Expression levels of extracellular signal-regulated protein kinase (ERK), phospho (p)-ERK, p-p38, p38, c-jun N-terminal kinase (JNK), and pJNK. (B, D, F) Quantification of (A, C, E). pERK/ERK, pP38/p38, and pJNK/JNK as a percentage of the LPS-treated alone group (set as 100%). α-Tubulin was used as the loading control. All data are presented as the mean ± SEM (*n* = 3). Statistical differences were analyzed using one-way ANOVA followed by Tukey statistical *post hoc* test. *p < 0.05, **p < 0.01, ***p < 0.001 vs. LPS-treated group, ^###^*p* < 0.001 vs. untreated control group.

The MAPK signaling pathway should be considered for the inhibition of neuroinflammation. We analyzed the phosphorylation of p38, ERK, and JNK, which are key players in the MAPK pathway, using western blotting analysis. Our results showed that HsAE reduced the phosphorylation of ERK by 79.54 ± 1.40 and 66.58 ± 1.08% at concentrations of 100 and 150 µg/mL, respectively (Figure 5(B)). Similarly, HsAE also significantly decreased the phosphorylation of p38 at concentrations of 25, 50, 100, and 150 µg/mL by 20.54, 17.44, 34.93, and 33.43%, respectively, compared to the LPS treatment group (Figure 5(D)). Remarkably, HsAE increased JNK phosphorylation and showed the opposite effect on ERK and p38 phosphorylation (Figure 5(F)).

### HsAE prevents neurotoxicity in LPS-stimulated microglial cells and activates microglia-induced N2a neuronal cells

**Figure 6.**
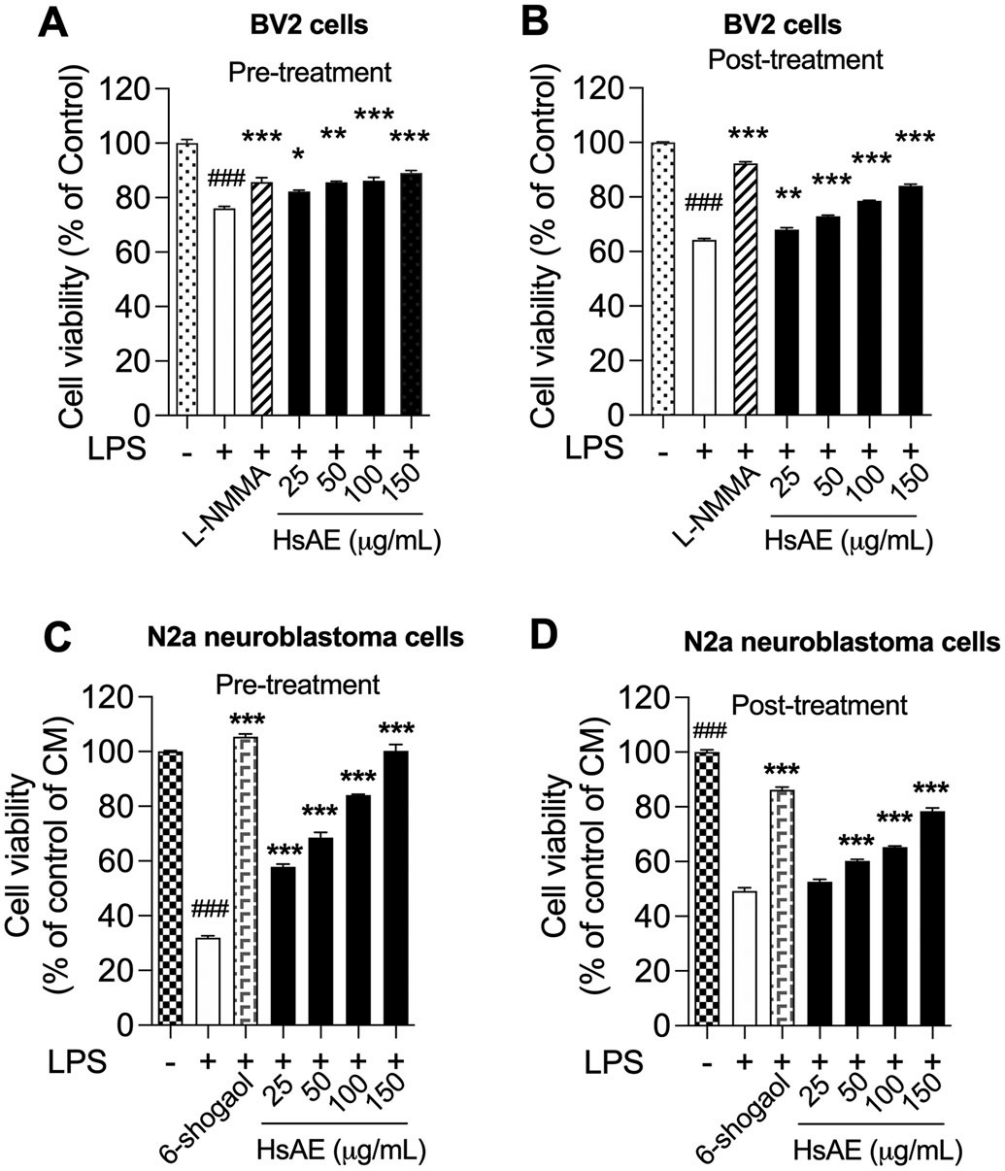
HsAE protects BV-2 cells from toxicity of LPS and N2a cells against LPS-stimulated apoptosis (A, B) Cytotoxic effects of pre- and post-treatment with HsAE at concentrations of 25, 50, 100, and 150 µg/mL for 30 min, followed by induction with LPS (100 ng/mL) for 24 h. (C, D) Cytoprotective effects of HsAE-treated BV-2 conditioned media in N2a cells after pre- and post-treatment. Cell viability is expressed as a percentage of the control group (set as 100%). All data are presented as the mean ± SEM (*n* = 3). Statistical differences were analyzed using one-way ANOVA followed by Tukey statistical *post hoc* test. *p < 0.05, **p < 0.01, ***p < 0.001 vs. LPS-treated group, ^###^*p* < 0.001 vs. untreated control group.

In LPS-stimulated BV-2 cells, the production of pro-inflammatory cytokines and mediators led to neuronal death, which also stimulated apoptosis of other neurons in the CNS. Neuroprotective effects of HsAE on LPS-induced neuronal cells were assessed by determining the cell viability. BV-2 cells were stimulated with 100 ng/mL LPS for 30 min before or after treatment with HsAE (25, 50, 100, or 150 µg/mL). LPS at a concentration of 100 ng/mL induced approximately 23.93 and 35.77% cell death during pre-treatment and post-treatment, respectively, which confirms that LPS is toxic to the BV-2 cell line. HsAE significantly increased the viability of BV-2 cells exposed to LPS, with a maximum effect observed at 150 µg/mL under both treatment conditions (Figure 6(A,B)).

N2a cell viability was reduced when treated with the CM of LPS, confirming that the CM of LPS alone induced significant cytotoxicity (Figure 6(C,D)). In the pre-treatment, the CM of HsAE increased the viability of cells by 25.91, 36.57, 52.13, and 68.26% at concentrations of 25, 50, 100, and 150 µg/mL, respectively, compared to the LPS treatment sample (Figure 6(C)). In the post-treatment, the CM of HsAE significantly increased the cell viability only at concentrations of 50, 100, and 150 µg/mL (Figure 6(D)).

## Discussion

Neuroinflammation is an inflammatory process involved in normal brain function that leads to neurodegeneration due to the innate and adaptive immune systems in the brain. Inflammation associated with AD progression may be induced by different cytokines and ILs (Calsolaro and Edison 2016). Microglia are the first and foremost form of active immune defense in the CNS. In the pathological condition of neuroinflammation, microglial cells are stimulated and their morphology changes. It triggers the release of inflammatory mediators, such as chemokines, cytokines, and cytotoxic molecules (such as COX-2, glutamate, and prostaglandins). Microglia can activate intracellular downstream signaling pathways involving MAPKs (Dragic et al. 2021). Extracellular stimuli, such as LPS, a component of gram-negative bacteria, can activate microglia and secrete pro-inflammatory mediators, such as NO, PGE_2_, IL-1β, IL-6, and TNF-α, which initiate several major cellular responses that contribute to the pathogenesis of neuroinflammation (Batista et al. 2019). Therefore, interventions to inhibit the production of these mediators are a promising approach for the prevention or treatment of neuroinflammation.

*H. serrata*, a genus of the family Lycopodiaceae, contains huperzine A as the active component. Lycopodium alkaloids from *H. serrata* are categorized into four classes: lycodine, lycopodine, fawcettimine, and miscellaneous (Liu et al. 2017). Various studies have reported its anti-inflammatory, anti-Alzheimer, antiapoptotic, and antioxidant effects (Ma et al. 2007; Vallejo et al. 2013). Huperzine A, a sesquiterpene alkaloid found naturally in *Huperzia* species and isolated from *H. serrata*, acts as an antioxidant and neuroprotective agent that contributes to the treatment of memory loss and mental impairment (Wang et al. 2008).

Anti-neuroinflammatory effects of HsAE and its molecular mechanisms in activated microglial cells have not yet been reported. More evidence is needed to evaluate the effectiveness of HsAE in treating neurodegenerative diseases and analyze its bioactive components, which enhance the anti-inflammatory potency. Thus, in this study, the anti-neuroinflammatory effects of HsAE and its bioactive components were investigated in LPS-stimulated microglial cells, which induce the inflammatory mediators.

To evaluate the anti-neuroinflammatory effects of HsAE, we used it to treat BV-2 cells that were overactivated by LPS stimulation, and the reduction of inflammatory markers was expected to prove the anti-inflammatory potential of HsAE against neuroinflammation. As shown in the results (see Figures 2–5), HsAE, the alkaloid fraction of HsE, drastically suppressed the inflammatory mediators, such as NO and pro-inflammatory cytokines (e.g., TNF-α, PGE2, IL-1β, IL-6), MAPK signaling, and related inflammatory factors (iNOS and COX-2) in LPS-stimulated microglial cells, indicating that HsAE could exert protective inflammatory effects by regulating the secretion or expression of inflammatory mediators. In BV-2 cells, LPS stimulation activated the inflammatory response, which was indicated by the secretion of inflammatory mediators (e.g., NO and PGE2) and pro-inflammatory cytokines (e.g., TNF-α, IL-1β, and IL-6). The secretion of inflammatory response products by reactive microglial cells is associated with MAPK signaling, which leads to neurotoxicity and neurodegeneration. LPS induces iNOS and TNF expression in microglial cells *via* the ERK1/2, p38 MAPK, and nuclear factor (NF)-κB signaling pathways (Jung et al. 2007). In the present study, HsAE effectively inhibited p38 and ERK1/2 MAPK phosphorylation. JNK phosphorylation showed the opposite trend, possibly due to another player, which requires further investigation. This finding suggests that LPS-induced microglial activation was mostly inhibited by HsAE *via* p38 and ERK1/2 MAPK phosphorylation signaling. Interestingly, pre-treatment with HsAE inhibited the inflammatory response in a dose-dependent manner; however, in the post-treatment setting, HsAE did not affect IL-1β secretion. Among the cytokines, IL-1β was found to be unchanged when HsAE was added after LPS stimulation, implying that the inflammatory signal was still sent out to alert the immune system. This result suggests the greater application of pretreatment as a prophylactic strategy (Figure 3). After LPS treatment, HsAE partially inhibited the inflammatory response, indicating that it may affect the inner link in the signaling chain, possibly the NF-κB or MAPK pathway.

HsAE acts as a potent acetylcholinesterase inhibitor that increases the levels of memory neurotransmitters in the brain and prevents beta-amyloid-induced oxidative stress (Arya et al. 2021). Our data showed that both pre- and post-treatment with HsAE could rescue LPS-stimulated BV-2 cells (Figure 6). Moreover, the surrounding normal N2a cells were also saved despite the hazardous impact of released inflammatory chemokines and cytokines in the CM. This is crucial not only for the inhibition of inflammation, but also for the protection of the brain from degradation.

Regarding the active agents in HsAE, we profiled the main components in the alkaloid fraction *via* chemical analysis, and found that huperzine A was the main active component at 7.35% (Dang et al. 2022). According to our previously reported results, HsAE increased the cholinergic transmission by inhibiting AChE and BuChE with IC_50_ values of 7.93 (5.43–10.98) µg/mL and 76.67 (64.78–91.84) µg/mL, compared to huperzine A with IC_50_ values of 0.082 µM (0.02 µg/mL) and 74.43 µM (18.04 µg/mL) for AChE and BuChE, respectively (Wang et al. 2006; Hoai et al. 2020). As shown in Table 2, the NO reduction efficacy of huperzine A and HsAE was as follows: pre-treatment, IC_50_ value of HsAE, 99.79 µg/mL and IC_50_ value of huperzine A, 38.04 µg/mL; and post-treatment, IC_50_ value of HsAE, 92.40 µg/mL and IC_50_ value of huperzine A, 40.23 µg/mL. UPLC-MS/MS analysis also showed the presence of other alkaloids, such as huperzine B and huperzine M, in HsAE. Our results imply that HsAE, the total alkaloid component of *H. serrata*, may ameliorate neurodegeneration by interfering with the initial inflammatory phase *via* activation of the CNS immunocompetent cells, such as microglia, indicating that HsAE may be a good source for developing new anti-neuroinflammatory agents.

## Conclusions

In this study, we investigated HsAE as a potential anti-neuroinflammatory agent. The cytotoxicity data revealed the safety of HsAE in BV-2 cells up to a concentration of 150 µg/mL. At all tested concentrations, HsAE inhibited the release of NO and cytokines, such as TNF-α, IL-6, IL-1β, and PGE2. At the intracellular level, HsAE suppressed the expression levels of iNOS and COX-2 *via* the regulation of the MAPK pathways, including ERK and p38 pathways. Pre-treatment of BV-2 cells with HsAE resulted in a more robust and comprehensive anti-neuroinflammatory effect, indicating the potential of HsAE as a prophylactic and therapeutic agent against neuroinflammation. Moreover, HsAE is a potent agent to rescue microglia and normal cells from death, which are essential for the treatment of dementia. Therefore, HsAE shows potential for the management of dementia owing to its anti-neuroinflammatory and neuroprotective effects.
